# Resonant Photonic Biosensors with Polarization-Based Multiparametric Discrimination in Each Channel

**DOI:** 10.3390/s110201476

**Published:** 2011-01-26

**Authors:** Robert Magnusson, Debra Wawro, Shelby Zimmerman, Yiwu Ding

**Affiliations:** 1 Department of Electrical Engineering, University of Texas at Arlington, Box 19016, Arlington, TX 76019, USA; 2 Resonant Sensors Incorporated, 416 Yates Street, 518 NH, Arlington, TX 76010, USA; E-Mails: wawro@resonantsensors.com (D.W.); zimmerman@resonantsensors.com (S.Z.); dingyw99@yahoo.com (Y.D.)

**Keywords:** guided-mode resonance sensors, leaky-mode resonance, periodic elements, biosensors, label-free sensors

## Abstract

In this paper, we describe guided-mode resonance biochemical sensor technology. We briefly discuss sensor fabrication and show measured binding dynamics for example biomaterials in use in our laboratories. We then turn our attention to a particularly powerful attribute of this technology not possessed by competing methods. This attribute is the facile generation of multiple resonance peaks at an identical physical location on the sensor surface. These peaks respond uniquely to the biomolecular event, thereby enriching the data set available for event quantification. The peaks result from individual, polarization-dependent resonant leaky modes that are the foundation of this technology. Thus, by modeling the binding event and fitting to a rigorous electromagnetic formalism, we can determine individual attributes of the biolayer and its surroundings and avoid a separate reference site for background monitoring. Examples provide dual-polarization quantification of biotin binding to a silane-coated sensor as well as binding of the cancer biomarker protein calreticulin to its monoclonal IgG capture antibody. Finally, we present dual-polarization resonance response for poly (allylamine hydrochloride) binding to the sensor with corresponding results of backfitting to a simple model; this differentiates the contributions from biolayer adhesion and background changes.

## 1. Introduction

There is an urgent, growing need to develop cost-effective, reliable sensor technologies for a host of applications in homeland security, biomedicine, drug development, food safety and environmental monitoring. For example, increased concerns with the effects of toxins (mutagens, bacteria and hazardous chemicals) in the environment require monitoring of pollutant levels at industrial, wastewater, urban and agricultural sites. Portable, low-cost, quick-responding sensors with sufficient sensitivity and reliability are needed for continuous, accurate monitoring of environmental pollution in the field. Most biosensor technologies currently available employ fluorescent or absorption labeling to register a specific biomolecular reaction. For reasons of expense and expediency, there is increasing demand for improved sensor techniques that do not require labeling. They must allow a wide range of molecules to be selectively screened with minimal assay development using readily available antibody-antigen, nucleic acids and other highly selective biomaterials. For environmental monitoring, miniature, self-contained, battery- or solar-powered sensors may be required where other sensor probes may not be useable. These sensors may form sensor networks using wireless modules for data transmission, communication and alarm. Photonic sensor technologies are advantageous as they are immune to outside electromagnetic interference, permit compact format and some types enable effective light input and output. With aim to meet many of these requirements, in this paper we provide sensors based on a unique photonic resonance concept. These sensors are broadly applicable in biomedicine and other application areas; our focus presently is on biosensors.

Resonant leaky modes can be induced on dielectric, semiconductor and metallic periodic layers patterned in one or two dimensions. Among potential applications are ultrasensitive biosensors that can be realized in a wide range of geometries and system architectures. Thus, we invented and implemented highly accurate, label-free guided-mode resonance (GMR) biosensors that are being commercialized. In 1992, Magnusson and Wang [1] suggested application of the GMR effect for sensor applications and disclosed GMR filters that were tunable on variation in resonance structure parameters including thickness and refractive index [2]. Tibuleac *et al.* and Wawro *et al.* presented new GMR biosensor embodiments as well as new possible applications of these sensors when integrated with optical fibers [3,4]. Following this work, Kikuta *et al.* [5], Cunningham *et al.* [6,7] and Fang *et al.* [8,9] also discussed the use of these resonant elements as biosensors.

The GMR sensor is based on the high parametric sensitivity inherent in the fundamental resonance effect. As an attaching biomolecular layer changes the parameters of the resonance element, the resonance frequency (wavelength) changes. A target analyte interacting with a bio-selective layer on the sensor can thus be identified without additional processing or use of foreign tags. As the fundamental GMR element is a superior sensor with promising commercial applications, the interest in this technology has skyrocketed worldwide with numerous attendant publications appearing. Additional representative example papers [10–13] and book chapters [14,15] showcase this interest.

A great variety of optical sensors for bio- and chemical detection has been reported in the literature. Key label-free sensor technologies include surface-plasmon resonance sensors, MEMS-based sensors, nano-sensors (rods and particles), resonant mirrors, Bragg grating sensors, waveguide sensors, waveguide interferometric sensors, ellipsometry and grating coupled sensors [16–19]. Other methods include immunomagnetic separation, polymerase chain reaction and standard immunoassay approaches that incorporate fluorescent, absorptive, radioactive and luminescent labels [18,19]. Although dramatically different in concept and function, the surface-plasmon resonance (SPR) sensor [16,17] comes closest in features and operation to the GMR sensor applied in this work. The term surface plasmon (SP) refers to an electromagnetic field charge-density oscillation that can occur at the interface between a conductor and a dielectric (e.g., gold/glass interface). An SP mode can be resonantly excited by TM-polarized incident light but not TE-polarized light. Phase matching occurs by employing a metallized diffraction grating or by using total internal reflection from a high-index material such as in prism coupling or from a guided wave in an optical fiber. When an SPR surface wave is excited, an absorption minimum occurs in a specific wavelength band. While angular and spectral sensitivity is very high for SPR sensors, the resonance linewidth is rather large. Since only a single polarization (TM) can physically be used for detection, changes in refractive index and biolayer attachments cannot simultaneously be resolved in one measurement. This is a particularly significant problem in portable diagnostic applications where thermal variations are probable.

## 2. Experimental Section

### 2.1. Guided-Mode Resonance Biosensors: Background

This research addresses the development of compact, high-performance GMR biosensors [1–4]. The heart of the sensor is a periodic dielectric waveguide (sometimes referred to as photonic crystal) in which resonant leaky modes are excited by an incident optical wave [20–33]. Most commonly, the input light is efficiently reflected in a narrow spectral band whose central wavelength is highly sensitive to chemical reactions occurring at the surface of the sensor element. In high-index media, such as silicon, interesting mode-mixing effects enable operation in narrow spectral or angular transmission bands [34,35]. This mode of operation is also of interest for sensor development, although it is not specifically addressed here.

The sensor’s operating spectral region, neighboring the resonance wavelength λ, is conveniently determined by the chosen grating period Λ. Interaction of a target analyte with a bio-selective layer on the sensor surface yields measurable spectral/angular shifts that directly identify the binding event without additional processing or foreign tags. A bio-selective layer (such as antibodies) is incorporated on the sensor surface to impart specificity during operation. Sensor designs responsive to thickness changes from the nanoscale (<∼10^−2^ nm) to several μm have been analyzed. These studies indicate that the proposed sensor technology can be used to detect binding events at the molecular level as well as bacterial analytes with micron-scale dimensions.

The fact that GMR sensors operate without foreign tags or labels is very significant, enabling expedient sample preparation in practice. This attribute implies detection methods that do not require the use of chemical indicators for read-out, such as fluorescent, luminescent or radioactive tags. The key point is that with GMR sensors, the reaction is optically monitored directly. In contrast, in label-based methodologies, the monitoring proceeds via the label, for example, by measuring emitted radiation under fluorescence.

**Figure 1.**
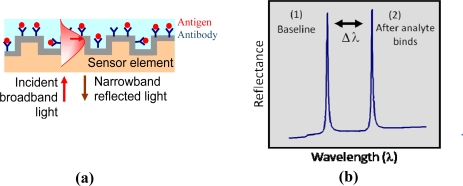
**(a)** Schematic of a guided-mode resonance sensor. **(b)** Binding events occurring at the sensor surface produce resonance-peak shifts that can be tracked in real time.

When a broadband light source illuminates GMR sensors, a specific wavelength of light is reflected or transmitted at a particular angle. Figure 1(a) schematically shows the binding interaction of an immobilized receptor with an analyte; it can be monitored, without use of chemical tags, by following the corresponding resonance wavelength shift with a spectrometer as denoted in Figure 1(b). Since the resonance layer is polarization-sensitive, separate resonance peaks occur for incident TE (electric vector normal to the plane of incidence) and TM (magnetic vector normal to the plane of incidence) polarization states. The sensor element can be prepared with standard surface chemistries to covalently attach a selective detection layer (such as antibodies or aptamers). The sensor is multifunctional as only the sensitizing surface layer needs to be chemically altered to detect different species. This sensor technology is broadly applicable to medical diagnostics, drug discovery and development, industrial process control and environmental monitoring.

**Figure 2.**
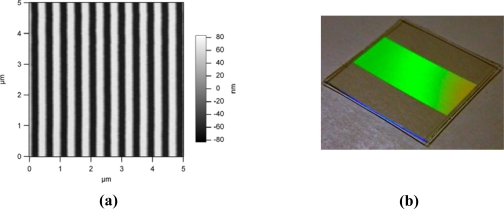
Submicron resonant grating. **(a)** Atomic force microscope (AFM) picture of a ∼500-nm period grating contact printed in an optical polymer. **(b)** A picture of a submicron molded grating. The grating is coated with a thin high-index layer (TiO_2_ or HfO_2_) to realize a GMR sensor element.

We fabricate GMR filters and sensors in dielectric media such as moldable polymers, fused silica, silicon dioxide, hafnium dioxide, silicon nitride and other materials. In particular, in this paper, the GMR biosensors embody a single-layer filter design fabricated using low-cost submicron molding methods. We utilize polymers imprinted with submicron grating patterns (∼500 nm grating periods) and coated with a high-index dielectric material (such as TiO_2_ or HfO_2_) to realize resonant sensors. Figure 2 shows an example of a GMR sensor. These sensors are designed to operate in the near-IR wavelength range (700–900 nm), where most biochemical materials have minimal absorption.

**Figure 3.**
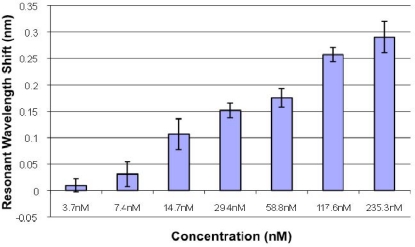
Sensor response (TM-polarization) for detection of TNF in buffer solution with immobilized anti-TNF antibodies bound to the sensor surface.

Numerous characterization experiments have been performed for a variety of model biomaterials utilizing GMR sensors and the Vides™ bioassay spectroscopic reader system developed by Resonant Sensors Incorporated. Figure 3 illustrates the spectral resonance peak shift due to the binding of tumor necrosis factor alpha (TNFα) in various concentrations. TNF is an inflammatory mediator that is often measured in disease research and drug discovery applications. To impart selectivity, an antibody layer that specifically binds to TNF is immobilized on the sensor surface by adsorption. Unbound sites are subsequently blocked using a commercially available, immunoassay-blocking buffer. The standard high-TNFα (Rat, 17 kDa, R&D Systems) stock solution is set at 4 μg/mL (235 nM). Stepwise two-fold dilutions are prepared using phosphate buffered saline (PBS) to obtain the desired concentrations. PBS is used as a reference blank and subtracted from the results. Results are repeated in at least triplicates and averaged. The TM polarization resonance peak is tracked in this particular experiment, and binding is monitored for 1 hour.

### 2.2. Dual Polarization GMR Sensor

**Figure 4.**
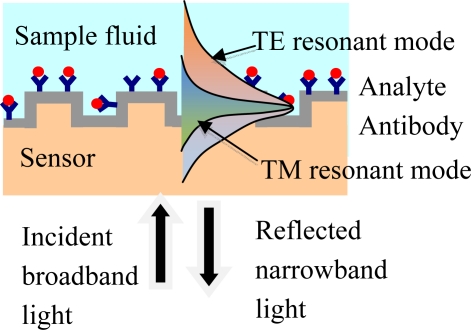
Schematic of a single channel in a label-free GMR sensor system operating in reflection mode. The collimated beam from a broadband source is incident on the sensor at normal incidence. The reflected spectral response is monitored in real time with an optical spectrum analyzer. As binding events occur at the sensor surface, resonance peak changes can be tracked. The resonance occurs at different wavelengths or angles for TE and TM polarization states of the input light, enabling high-accuracy detection.

Polarization is a fundamental property of light. As a beam of light can possess arbitrary polarization states, the incident beam polarization state can be engineered cost-effectively to improve sensor performance provided that the sensor is physically capable of responding to such states. The GMR sensor has this capability since the resonance response is sensitive to the incident light polarization. Thus separate resonance peaks occur for incident TE and TM polarization states. This property provides enriched data sets useful for increasing detection accuracy in a given sensor element. The incident light excites photonic surface states shown as TE and TM modes. As schematically indicated in Figure 4, these modes interact differently with the surrounding media, enabling the polarization-based differentiation. The important point is that separate reference channels are not required to distinguish, for example, thermal background variations during data acquisition. This improves accuracy and reduces cost. Sensors arrays based on other concepts may apply a significant fraction of their sensor elements as reference monitors, which is not an efficient use of the chip.

## 3. Results and Discussion

### 3.1. Quantification of Biotin Binding

**Figure 5.**
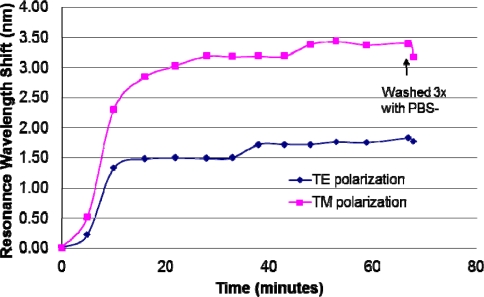
Illustration of the use of GMR sensor polarization diversity to quantify biotin binding (0.5 mg/mL Sulfo-NHS-LC-Biotin) to a silane-coated sensor surface. The molecular attachment event is monitored as function of time. Results for both TE and TM polarizations are shown. The results are consistent and exhibit the differing sensitivities associated with differing polarizations. At the end of the binding, any loose or unbound biotin is rinsed away in PBS-Tween solution.

**Figure 6.**
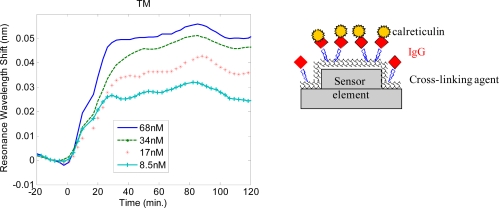
Resonance peak wavelength shift as a function of time for calreticulin binding to its matched IgG antibody. Solutions are monitored with calreticulin concentration range of 8.5nM to 68nM. A PBS blank is used as a reference and subtracted from the data. At t = 0, the calreticulin solutions are introduced to the sensor element and monitored in real time for 80 minutes. At t = 80 minutes, the calreticulin solution is removed and the sensor element is washed thoroughly with PBS/Tween and monitored for another 40 minutes to establish a post-binding baseline. Results are repeated in triplicate and averaged.

Using dual polarization data collection, Figure 5 illustrates how the binding of the foundational attachment chemistries (such as silane, antibodies and biotin-avidin layers) can also be monitored in real time. When implemented in a flow-cell geometry, this approach can be used to monitor the full cycle of molecular binding dynamics to determine association and disassociation rates for applications such as proteomics. Since the present work is targeted at concentration analysis, we implement the GMR sensor in a static micro-well format. Monitoring of the association dynamics (such as shown in Figures 5 and 6) is used to determine when the reaction has stabilized to an approximate equilibrium. By monitoring the quality and uniformity of the functionalization chemistries in an assay, repeatability and accuracy from well to well can increase. In this example, a N-Hydroxysulfosuccinimide ester of biotin is deposited on a silane-coated sensor element. We use a biotin with a long chain spacer arm attached (Sulfo-NHS-LC-Biotin) that reacts efficiently with primary amines (such as silane groups) on the sensor surface. This Sulfo-NHS-LC-Biotin analyte has a molecular weight of 557 Da. Note that there are inherently separate peaks for each polarization (TE and TM) that shift in response to the reaction. This distinguishing feature provides two concurrent sets of data that can be used to distinguish background index/density changes from the targeted antigen binding interactions, thus increasing detection accuracy and reducing false positive readings [36]. In this experiment, the TE resonance occurs near ∼780 nm and the TM resonance occurs near ∼795 nm. However, it is the relative peak shift that is most important, not the absolute resonance locations.

### 3.2. Quantification of Cancer Biomarkers

**Figure 7.**
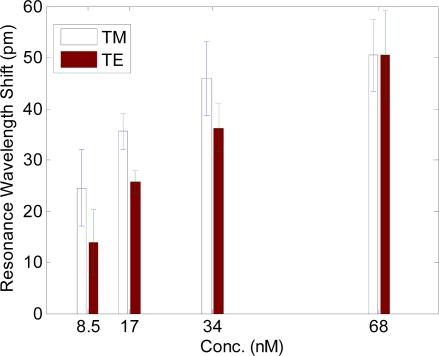
Dual polarization resonance response for calreticulin binding to the activated sensor element. These results are gathered after a post-PBS/Tween wash, indicating bound calreticulin remaining on the sensor surface. A PBS blank is used as a reference and subtracted from the data. Results are repeated in duplicate and averaged. Error bars shown indicate estimated uncertainty in each measurement.

Prior research has identified potential indicators and screening targets for the early detection and diagnosis of ovarian carcinoma to monitor presymptomatic aspects of the disease and disease progression [37,38]. While there are currently no clinically established diagnostic tools using urinalysis or seranalysis, experimentally and clinically identified biomarker proteins include calreticulin, ryanodine receptor 3 and others. We have conducted experiments investigating the detection of several of these biomarkers, including calreticulin. Calreticulin has a molecular weight of ∼46 kDa and has an elliptical shape ∼30 nm long and ∼2.4 nm wide [39]. For detection of the biomarker protein calreticulin, the capture antibody used is a specific monoclonal IgG antibody (anti-calreticulin). The sensor plates are initially coated with a commercially available silane (3-Aminopropyltriethoxysilane) that provides means to covalently bond the calreticulin antibody to the sensor surface. It is chemically attached to the silane sensor surface using the homobifunctional cross-linking agent disuccinimidyl suberate (DSS). The sensor element is then blocked with a 3% milk solution to minimize non-specific binding. Next, the plate is aspirated and washed with PBS/Tween in preparation for use. A known standard concentration of 68nM (3.75 μg /mL) calreticulin is used as the high standard. Stepwise two-fold dilutions of calreticulin are prepared using PBS to obtain the desired concentrations. PBS is used as a reference blank. A kinetic response of calreticulin binding to the antibody-coated sensor element is shown in Figure 6. The sensors are incubated in calreticulin solution for 80 minutes and subsequently washed with PBS/Tween to remove unbound material. TE and TM resonance wavelength shifts are then recorded as shown in Figure 7. While the sensor operates in real time, the speed of detection is often limited by the biochemical binding dynamics, which can be affected by temperature, humidity and selective layer affinity.

### 3.3. Application to Determine Two Parameters Simultaneously

A GMR sensor layer that supports N modes in a given wavelength band exhibits N resonance peaks. Suppose we design the sensor to support the fundamental modes TE_0_ and TM_0_. Then, an unpolarized interrogating beam will generate corresponding separate resonance peaks. These resonance peaks shift in response to the reaction, providing two sets of data. By backfitting this dual-peak response into our rigorous electromagnetic coupled-wave analysis [40] codes, we can determine two unknowns. This powerful backfitting approach can be used to distinguish background index changes, such as those that might occur due to thermal or sample background changes, from attaching biolayers; this provides the potential to significantly reduce false positives and testing errors.

**Figure 8.**
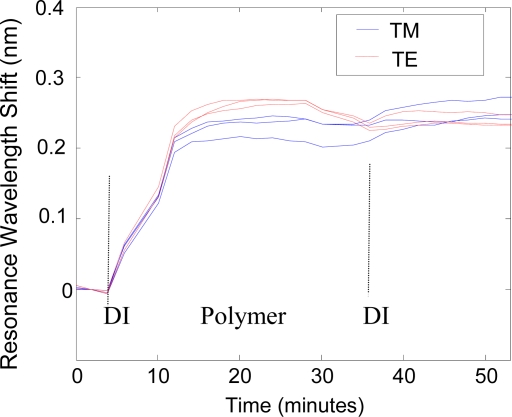
Dual polarization resonance response for poly (allylamine hydrochloride) binding to the sensor showing resonance wavelength shift as a function of time. This medium has molecular weight of 56 kDa; we use a concentration of 1.3mg/ml and pH 9.0.

We now take a concrete example of dual-peak resonance detection applying TE_0_ and TM_0_ leaky modes and backfit to a mathematical model. Using our in-house rigorous electromagnetic codes, we can accurately calculate the surface and bulk-change contributions to the resonance shifts during a biochemical binding experiment. Thus, we calculate and map the TE and TM resonance peak shifts over a range of added biolayer thicknesses (0 to 50 nm) and background index variations (n = 1.33 to n = 1.5). The resulting look-up table is applied to match the corresponding detection layer and background index when the two resonance peak shifts are known. We use the ionic polymer poly (allylamine hydrochloride) [41,42] to study binding interactions that involve biolayer adhesion and associated thickness change at the sensor surface. A baseline is taken with de-ionized (DI) water, and then the DI water is removed by pipetting (while the measurement is briefly paused), and the ionic polymer is dispensed on the sensor surface. Two resonance peaks are tracked as the ionic polymer attaches a monolayer of material as shown in Figure 8. After the polymer saturates, the sensor measurement is paused and the element is rinsed with DI water to remove any unbound polymer.

**Figure 9.**
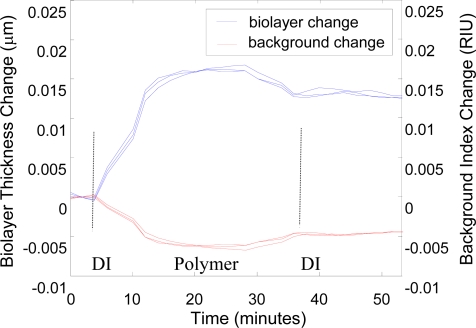
Results of backfitting to a simple model, thereby differentiating contributions from biolayer adhesion and background changes. Thus, we estimate the final adhered layer thickness to be ∼15 nm as noted by the scale on the left side of the figure.

A final measurement is made in DI water. This data is fitted to a model assuming biolayer refractive index of 1.4 taking layer thickness and background liquid index as unknowns. The results in Figure 9 show that the binding of the polymer layer to the sensor surface generates the most of the peak shift response. There is a slight drift in the background index as the fluid incubates on the sensor element, contributing to the sensor response. Viewed by itself, the raw peak shift data in Figure 8 does not distinguish between surface changes and bulk changes; this method differentiates the contributions. The fitted background drift is partially attributed to thermal changes in the liquid during the measurement and partly to imperfect model assumptions (such as polymer layer index). Improvements to the backfit model will further distinguish these contributions.

## 4. Conclusions

In summary, the biosensor technology discussed in this paper is based on simple subwavelength waveguide gratings that are easy to fabricate and amenable to economic mass production. When these sensors are illuminated with a light source, the sensor undergoes a guided-mode resonance such that a specific wavelength of light is reflected (with a corresponding transmission minimum) at a particular angle. Interaction of a target analyte with a biochemical layer on the sensor surface yields measurable spectral shifts that directly identify the binding event without additional processing or foreign tags. The dielectric sensor chips are prepared with standard surface chemistries (such as silane) to covalently attach commercially available and validated specific antibody, aptamer or peptide layers. Commercial blocking agents are used to minimize non-specific binding effects in non-ideal backgrounds such as serum, food and environmental liquids. As the binding assay begins, the analyte binds to the detection layer target and a change in the resonance wavelength is measured with a spectrum analyzer. The amount of wavelength shift can be directly correlated to the quantity of analyte in a fluid or gas. The sensor is multifunctional as only the sensitizing surface layer needs to be chemically altered to detect different species.

In this contribution, we have specifically emphasized polarization-based parametric discrimination. Our resonant sensors can be designed to support two or more leaky modes in the spectral band of interest. These modes can be directly addressed with the interrogating beam of light and resonate in their respective polarization states. This property provides enriched data sets that can be used to calibrate simultaneously for variations such as temperature or sample background density, in the same identical sensor spot, increasing detection accuracy and reducing probability of false readings. Indeed, concurrent, co-localized data acquisition via such polarization and modal diversity eliminates errors associated with use of separate reference sites. GMR sensors can be arrayed into high-density (∼10,000 sensors/cm^2^) platforms that are easily interrogated. Separate input/output waveguides to each sensor element are not required. Typical limits of detection are in the pM range. Therefore, this sensor technology will find increasing application in medical diagnostics, drug development, industrial process control, genomics and environmental monitoring.
